# Using Summary Statistics to Model Multiplicative Combinations of Initially Analyzed Phenotypes With a Flexible Choice of Covariates

**DOI:** 10.3389/fgene.2021.745901

**Published:** 2021-10-12

**Authors:** Jack M. Wolf, Jason Westra, Nathan Tintle

**Affiliations:** ^1^ Division of Biostatistics, School of Public Health, University of Minnesota, Minneapolis, MN, United States; ^2^ Department of Mathematics, Computer Science, and Statistics, Dordt University, Sioux Center, IA, United States; ^3^ Department of Population Health Nursing Science, College of Nursing, University of Illinois Chicago, Chicago, IL, United States

**Keywords:** summary statistics, covariate adjustment, linear models, phenotype, multiplication

## Abstract

While the promise of electronic medical record and biobank data is large, major questions remain about patient privacy, computational hurdles, and data access. One promising area of recent development is pre-computing non-individually identifiable summary statistics to be made publicly available for exploration and downstream analysis. In this manuscript we demonstrate how to utilize pre-computed linear association statistics between individual genetic variants and phenotypes to infer genetic relationships between products of phenotypes (e.g., ratios; logical combinations of binary phenotypes using “and” and “or”) with customized covariate choices. We propose a method to approximate covariate adjusted linear models for products and logical combinations of phenotypes using only pre-computed summary statistics. We evaluate our method’s accuracy through several simulation studies and an application modeling ratios of fatty acids using data from the Framingham Heart Study. These studies show consistent ability to recapitulate analysis results performed on individual level data including maintenance of the Type I error rate, power, and effect size estimates. An implementation of this proposed method is available in the publicly available R package pcsstools.

## 1 Introduction

Researchers now have readily available access to massive quantities of genotypic and phenotypic data (Cox, 2018; Simell et al., 2019). For example, *via* the Electronic Medical Records and Genomics {eMERGE Network^1^, the UKBiobank (Bycroft et al., 2018) other initiatives and repositories [e.g., 23andMe, MGI^2^ (Gagliano Taliun et al., 2020), FINRISK, CHOP (Diogo et al., 2018), among others]}, researchers can access a wide variety of phenotypic and genomics data on hundreds of thousands of individuals. However, important questions remain about how to best leverage these repositories. For example, the size of biobank datasets makes it challenging to transfer, store, and analyze data locally. While cloud computing minimizes some of these issues, it brings its own challenges related to cost (storage and computation), transfer, and access. Furthermore, data security and privacy issues are of paramount importance throughout all aspects of the data access, storage, and analysis pipeline (Jones et al., 2012; Heatherly, 2016; Simell et al., 2019).

A key innovation in this field is pre-computing non-individually identifiable summary statistics on biobank data and maximizing access to this data (Pasaniuc and Price, 2017). For example, GeneAtlas provides basic summary statistics for simple linear regression models of single nucleotide variants (SNVs) with 1,000s of available phenotypic variables across hundreds of thousands of individuals in the UK Biobank (Canela-Xandri et al., 2018), which also provides access to phenotype-phenotype correlations, single nucleotide polymorphism (SNP) minor allele frequencies (MAFs) and Hardy Weinberg Equilibrium (HWE) *p*-values. Likewise, PheWeb^3^ is a software toolkit which provides access to UK Biobank and Michigan Genomics Initiative data *via* a series of easy-to-navigate visualization and summary tools (Gagliano Taliun et al., 2020). Others (e.g., The Lee Lab for Statistical Genetics and Data Science^4^) simply provide access to sets of pre-computed summary statistics (PCSS) from large datasets. These resources mitigate many of the privacy and security concerns mentioned above since no individual participant data (IPD) is shared. In addition, the size of these repositories are only fractions of the size of IPD, making transfer and storage of the data much more efficient. Finally, pre-computing these summary statistics alleviates much of the computational burden on researchers who would otherwise have to calculate this information on their own. Despite these advantages, significant limitations currently exist when using these repositories of PCSS.

For example, researchers may want to modify a phenotype with available PCSS to one that is of greater clinical interest or use different sets of covariates than those considered in pre-computed analyses. Recent work is beginning to address these limitations. In two recent papers by our group (Gasdaska et al., 2019; Wolf et al., 2020), we demonstrated how to use standard PCSS (only means, variances, and correlations of all predictors and responses) to calculate the coefficients and standard errors for the linear model for a linear combination of phenotypes with an arbitrary set of covariates. This can then be used to perform Principal Component Analysis (PCA) on a set of phenotypes since principal component scores are just linear combinations with weights derived from the phenotype covariance matrix. Further, we demonstrated that if the phenotype correlation matrix is not available, we can use the correlation of test statistics for each phenotype across all genetic markers in its place with little loss of efficiency. These innovations mean that researchers can, using only PCSS, select the unique set of covariates they wish to adjust for and model a linear combination of phenotypes.

Importantly, these two approaches which require a priori specification of a phenotype of clinical interest, contrast to other recently developed methods which jointly and simultaneously analyze multiple phenotypes (Dutta et al., 2019a,b; Guo and Wu, 2019; Li et al., 2020; Ray and Boehnke, 2018) without an explicit characterization of the relationship between the phenotypes. These joint phenotype tests aim to simultaneously analyze multiple phenotypes while satisfying statistical objectives such as maximizing power under certain conditions. Furthermore, some of these approaches (Ray and Boehnke, 2018; Guo and Wu, 2019) do so using PCSS readily available from existing repositories.

Currently, our group’s methods for using PCSS to analyze modified phenotypes with flexible covariate choices are limited to PCA and choosing a phenotype that is a linear combination of the phenotypes for which PCSS are available. Another meaningful way to combine phenotypes is through multiplication. That is, several phenotypes of interest can be viewed as multiplicative combinations of other phenotypes for which PCSS may be available. Examples of note include fatty acid conversion ratios (Kalsbeek et al., 2018) and the body mass index (Justice et al., 2017). Additionally, products of binary phenotypes can be interpreted as logical “and” and “or” statements (e.g., a phenotype ***y*** that is defined as “***y***_1_ or ***y***_2_”). Various medical conditions are defined through logical combinations of various phenotypes. For example, coding ischemic strokes based on the union and intersection of various stroke subtypes (von Berg et al., 2020).

In this manuscript, we demonstrate how to analyze modified phenotypes which are multiplicative combinations of an arbitrarily large number of phenotypes for which PCSS are available. We also demonstrate how to flexibly adjust for covariates in these modified phenotype models. Importantly, we also show how the multiplication of phenotypes, when applied to binary phenotypes, allows for logical combination of phenotypes. After presenting a mathematical framework for the method, we validate the method using comprehensive simulations and demonstrate the method on real data from the Framingham Heart Study.

## 2 Methods

Consider the *m* phenotypes ***y***_1_, …, ***y***_*m*_ where each is an *n* × 1 vector of measures across *n* subjects and the *n* × *p* design matrix ***X*** = (***x***_1_, …, ***x***_*p*_) which consists of variables including genotypic information, covariates, and an intercept column. Moreover, let ***w***_*m*_ = ***y***_1_ ***y***_2_⋯ ***y***_*m*_ denote the pairwise Hadamard product of all *m* phenotypes for each subject. Our aim is to approximate the coefficients and standard errors of the covariate adjusted linear regression model for the product of *m* phenotypes: $w_{m}=X\hat{\beta }+$ ϵ using only readily available PCSS.

### 2.1 Assumed Pre-Computed Summary Statistics and Information

**FIGURE 1:**
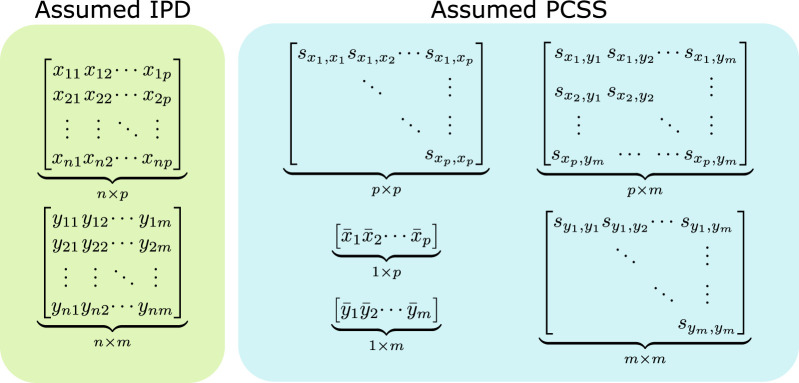
Data assumed when modeling using individual participant data (IPD) and when using pre-computed summary statistics (PCSS) to model a product of *m* phenotypes (***y***_1_, …, ***y***_*m*_) as a linear function of *p* covariates (***x***_1_, …, ***x***_*m*_). While modeling using IPD requires *n* × (*p* + *m*) points of data, using PCSS only requires *p* ^2^ + *pm* + *m* ^2^ + *p* + *m* values, which is far less when *n* is moderately large compared to *p* and *m*. All of these PCSS are readily available in existing PCSS repositories, or can be derived or approximated from other PCSS.

We assume knowledge of the following PCSS: the means of every predictor (e.g., SNPs and covariates), the means of every phenotype, and the full variance-covariance matrix of all predictors and phenotypes (i.e., $s_{x_{j},y_{k}}$ , $s_{x_{j},x_{i}}$ and $s_{y_{k},y_{l}}$ for any *i*, *j*, *k*, *l* where 1 ≤ *i*, *j* ≤ *p* and 1 ≤ *k*, *l* ≤ *m*). These are all readily available in standard PCSS repositories. We also assume to know the marginal distribution that each predictor and phenotype follows (e.g., binomial, normal, etc.). Figure 1 displays the assumed information when modeling *via* both IPD and PCSS.

However, if some summary statistics are unknown, they may be able to be derived or approximated. For example, SNPs distributed in HWE can have their mean and variance approximated through a binomial distribution given the MAF. Furthermore, the covariance of a genetic variant and a non-genetic variable is calculated as the single-marker slope coefficient (for the model with the non-genetic variable as the response and the genetic variant as the predictor) divided by the variance of the genetic variant. Other published papers (Kim et al., 2015; Zhu et al., 2015) have shown that the correlation of two traits can be approximated by the correlation of *Z* statistics of SNPs not associated with either trait. This approximation method is described in detail in Ray and Boehnke (2018). Two of our previous papers (Gasdaska et al., 2019; Wolf et al., 2020) have demonstrated the accuracy of these three methods through both simulation and real-data applications.

### 2.2 Linear Regression With Covariates Using Pre-Computed Summary Statistics

Given a response vector ***w***_*m*_ and design matrix ***X*** = (***x***_1_, …, ***x***_*p*_) which includes *p* variables including SNPs’ minor allele counts, covariates, and a possible intercept column, the normal error regression model $w_{m}=X\beta +ɛ$ where ϵ $∼N\left(0,\sigma^{2}I\right)$ has ordinary least squares estimate for β : $\hat{\beta }={\left(X^{\prime }X\right)}^{-1}X^{\prime }w_{m}$ with $\mathrm{Var}\left(\hat{\beta }\right)={\hat{\sigma }}^{2}{\left(X^{\prime }X\right)}^{-1}$ . In a recent paper (Wolf et al., 2020), we demonstrated how to calculate these values using only PCSS using the facts that:

$$X^{\prime }X=\left(n-1\right)S\left(X\right)+n\bar{x}{\bar{x}}^{\prime }$$ (1)

$$X^{\prime }w_{m}=\left(n-1\right){\left(s_{w_{m},x_{1}},\ldots ,s_{w_{m},x_{p}}\right)}^{\prime }+n{\bar{w}}_{m}\bar{x}$$ (2)

$$w_{m}^{\prime }w_{m}=\left(n-1\right)s_{w_{m}}^{2}+n{\bar{w}}_{m}^{2}$$ (3)

and

$${\hat{\sigma }}^{2}=\left(w_{m}^{\prime }w_{m}-{\hat{\beta }}^{\prime }X^{\prime }w_{m}\right)/\left(n-p\right)$$ (4)

where *S*(***X***) is the *p* × *p* variance-covariance matrix of the columns of the design matrix ***X***, $\bar{x}={\left({\bar{x}}_{1},\ldots ,{\bar{x}}_{p}\right)}^{\prime }$ is the *p* × 1 vector of column means of ***X***, ${\bar{w}}_{m}$ is the mean of ***w***_*m*_, and $s_{w_{m},x_{j}}$ is the sample covariance between ***w***_*m*_ and ***x***_*j*_.

With these methods in mind and assumed access to standard PCSS, in order to approximate $\hat{\beta }$ , and $\mathrm{Var}\left(\hat{\beta }\right)$ for this covariate adjusted multiple linear regression model, all that remains is to estimate ${\bar{w}}_{m}$ , $s_{w_{m}}^{2}$ , and $s_{x_{j},w_{m}}$ for each *j*. We will first demonstrate how to approximate these values using PCSS when *m* = 2 in Section 2.3.1 and later show how recursion can be used when *m* > 2 in Section 2.3.2.

### 2.3 Covariance Estimation

#### 2.3.1 Covariance Estimation With the Product of 2 Phenotypes

Let ***w***_2_ = ***y***_1_ ***y***_2_ be the pairwise Hadamard product of ***y***_1_ and ***y***_2_. Then, if ***x***_*j*_ represents an “intercept” column of the design matrix with all elements unity [i.e., if ***x***_*j*_ = (1,…,1)′], we set $s_{x_{j},w_{2}}=0$ . Otherwise, we proceed as follows: We first approximate the conditional mean and variance of ***y***_*k*_ given ***x***_*j*_ = *x* for *k* ∈ {1, 2} through a linear regression model as

$$g\left(y_{k}|x\right)=a_{kj}+b_{kj}x$$ (5)

and

$$h\left(y_{k}|x\right)=\left(n-1\right)\left(s_{y_{k}}^{2}-b_{kj}s_{x_{j},y_{k}}\right)/\left(n-2\right)$$ (6)

where $b_{kj}=s_{x_{j},y_{k}}/s_{x_{j}}^{2}$ and $a_{kj}={\bar{y}}_{k}-b_{kj}{\bar{x}}_{j}$ . We note that this conditional variance will be constant at any value of *x* following from the linear regression assumption of homoscedasticity.

Then, we calculate the sample partial correlation of ***y***_1_ and ***y***_2_ controlling for ***x***_*j*_:

$$r_{y_{1},y_{2}.x_{j}}=\frac{r_{y_{1},y_{2}}-r_{x_{j},y_{1}}r_{x_{j},y_{2}}}{\sqrt{\left(1-r_{x_{j},y_{1}}^{2}\right)\left(1-r_{x_{j},y_{2}}^{2}\right)}},$$ (7)

setting $r_{y_{1},y_{2}.x_{j}}=0$ if either $r_{x_{j},y_{1}}$ or $r_{x_{j},y_{2}}=1$ . As the expectation of the conditional correlation equals the partial correlation under the assumption of a multivariate linear relationship between (***y***_1_, ***y***_2_) and ***x***_*j*_ (Baba et al., 2004), we use the partial correlation as an estimate of the conditional correlation of ***y***_1_ and ***y***_2_ at all possible values of ***x***_*j*_. So, we approximate the covariance of ***y***_1_ and ***y***_2_ conditional on ***x***_*j*_:

$$h\left(y_{1},y_{2}|x\right)=r_{y_{1},y_{2}.x_{j}}\sqrt{h\left(y_{1}|x\right)h\left(y_{2}|x\right)}$$ (8)

These terms let us approximate the conditional mean of ***w***_2_ at a given value *x* of ***x***_*j*_:

$$g\left(w_{2}|x\right)=g\left(y_{1}|x\right)g\left(y_{2}|x\right)+h\left(y_{1},y_{2}|x\right)$$ (9)

Then, letting *f*_*j*_(*x*) be an assumed probability distribution/mass function for ***x***_*j*_ with support $S_{j}$ [e.g., if ***x***_*j*_ is a vector of minor allele counts with MAF *p*, letting $f_{j}\left(x\right)=(\begin{array}{c} 2\\ x \end{array})p^{x}{\left(1-p\right)}^{2-x}$ and $S_{j}=\left\{0,1,2\right\}$ ) we approximate the sample covariance of ***x***_*j*_ and ***w***_2_:

$$s_{x_{j},w_{2}}\approx \underset{x\in S_{j}}{\sum }f_{j}\left(x\right)\left(x-{\bar{x}}_{j}\right)g\left(w_{2}|x\right),$$ (10)

swapping the sums for integrals across the support when appropriate.

We calculate the sample mean of ***w***_2_ as

$${\bar{w}}_{2}={\bar{y}}_{1}{\bar{y}}_{2}+s_{y_{1},y_{2}}\left(n-1\right)/n$$ (11)

To approximate the variance, we first approximate the conditional variances of ***w***_2_ at all levels of ***x***_*j*_:

$$\begin{array}{cc} h\left(w_{2}|x\right)= & \;h\left(y_{1}|x\right)h\left(y_{2}|x\right)+g\left(y_{1}|x\right)h\left(y_{2}|x\right)+\\ & \;g\left(y_{2}|x\right)h\left(y_{1}|x\right)+g\left(w_{2}|x\right)h\left(y_{1},y_{2}|x\right) \end{array}$$ (12)

And then approximate the sample variance as:

$$s_{w_{2}}^{2}\approx \left(\underset{x\in S_{j}}{\sum }\left(nf_{j}\left(x\right)-1\right)h\left(w_{2}|x\right)+nf_{j}\left(x\right){\left(g\left(w_{2}|x\right)-{\bar{w}}_{2}\left.\right)\right)}^{2}\right)/\left(n-1\right)$$ (13)

once again swapping the sum for an integral across $S_{j}$ when appropriate. This approach leads to a different variance estimate for each predictor ***x***_*j*_. We treat the median of these estimates across each *j* as the estimated variance.

Hence, taking the means, variances, and pairwise covariances of ***x***_*j*_, ***y***_1_, and ***y***_2_ and a distributional assumption about ***x***_*j*_, we approximate the covariance of one variable (***x***_*j*_) with the product of the other two (***w***_2_ = ***y***_1_ ***y***_2_) as well as the product’s mean and variance.

Repeating this algorithm for each predictor ***x***_*j*_ and following the linear regression equations presented in Section 2.2 allows for calculation of covariate adjusted slope coefficients for the multiple regression model $w_{2}=X\hat{\beta }+$ ϵ as well as the standard errors of these slope estimates.

#### 2.3.2 Covariance Estimation With the Product of 3 or More Phenotypes

Regression models for larger products of phenotypes can also be approximated by applying the established method recursively: first estimating the covariance of ***x***_*j*_ and ***w***_2_, then leveraging the covariance of ***x***_*j*_ and ***w***_2_ and ***x***_*j*_ and ***y***_3_ to estimate the covariance of ***x***_*j*_ and ***w***_3_, and so forth. This recursion procedure is described in more detail in the appendix and software to carry it out is discussed in Section 2.8.

Let ***w***_*l*_ = ***y***_1_ ***y***_2_⋯ ***y***_*l*_ = ***w***_*l*−1_ ***y***_*l*_. In order to estimate $s_{x_{j},w_{l}}$ through our established method, we use ${\bar{x}}_{j}$ , ${\bar{y}}_{l}$ , ${\bar{w}}_{l-1}$ , $s_{x_{j}}^{2}$ , $s_{y_{l}}^{2}$ , $s_{w_{l-1}}^{2}$ , $s_{x_{j},y_{l}}$ , $s_{x_{j},w_{l-1}}$ , and $s_{w_{l-1},y_{l}}$ as inputs to the method described in Section 2.3.1. That is, replacing ***y***_1_ with ***w***_*l*−1_ and ***y***_2_ with ***y***_*l*_. While ${\bar{x}}_{j}$ , ${\bar{y}}_{l}$ , $s_{x_{j}}^{2}$ , $s_{y_{l}}^{2}$ , and $s_{x_{j},y_{l}}$ are assumed to be known, we must estimate $s_{x_{j},w_{l-1}}$ and $s_{w_{l-1},y_{l}}$ .

Continuation of the recursive process starting at ***w***_*l*−1_ and working down to ***w***_2_ will yield an estimate for $s_{x_{j},w_{l-1}}$ , or eventually the base case of $s_{x_{j},w_{2}}$ .

To approximate $s_{w_{l-1},y_{l}}$ , we re-express the term as $s_{y_{l},w_{l-2}y_{l-1}}$ and approximate the covariance of ***y***_*l*_ and ***w***_*l*−2_ ***y***_*l*−1_ through the method described in Section 2.3.1.

A diagram of the start of this recursion is displayed in Supplementary Figure S1.

This recursive estimation is impacted by the order in which the phenotypes are multiplied. So, any set of more than two phenotypes will render *m*!/2 possible ways to estimate the regression model through this method. Hence, we approximate the covariances and means using all permutations of ***y***_1_, …, ***y***_*m*_ unique up to the order of the first two terms as the order of our phenotypes, and take the median estimate of each term across all permutations as its predicted value.

### 2.4 Binary Phenotypes

Binary phenotypes present both new challenges to estimation and the opportunity to express logical combinations of phenotypes through products.

#### 2.4.1 Changes to Estimations

Our proposed method leverages several assumptions of the standard linear regression model in its approximations (namely those of homoscedasticity and linearity). As binary phenotypes notoriously violate both of these assumptions, we slightly adjust our approximation to better reflect this situation.

The covariance of two binary phenotypes is estimated using the same general framework as developed in Section 2.3.1. The only changes are to mean the variance estimates. All estimated means (conditional and otherwise) are initially estimated as proposed in Section 2.3.1 and then restricted to the open interval (0, 1) (e.g., letting *g*(*y*_*k*_|*x*) = *a*_*kj*_ + *b*_*kj*_ *x* if 0 < *a*_*kj*_ + *b*_*kj*_ *x* < 1, ϵ if *a*_*kj*_ + *b*_*kj*_ *x* ≤ 0, and 1 − ϵ if 1 ≤ *a*_*kj*_ + *b*_*kj*_ *x* for some small ϵ > 0).

Instead of estimating a phenotype’s conditional variance from a linear model’s residual variance, we estimate it as

$$h\left(y_{k}|x\right)=g\left(y_{k}|x\right)\left(1-g\left(y_{k}|x\right)\right)$$ (14)

Further, we calculate the product’s sample variance as

$$s_{w_{2}}^{2}={\bar{w}}_{2}\left(1-{\bar{w}}_{2}\right)n/\left(n-1\right)$$ (15)

#### 2.4.2 Products as Logical Combinations

Binary phenotypes are of particular importance because their products can be interpreted as logical combinations.

We can represent the logical conjunction ***y***_1_ ∧ ***y***_2_ (read as “***y***_1_ and ***y***_2_”) as the product ***y***_1_ ***y***_2_. Likewise, we express the logical disjunction ***y***_1_ ∨ ***y***_2_ (“***y***_1_ or ***y***_2_”) as **1**_*n*_ − ((**1**_*n*_ − ***y***_1_)(**1**_*n*_ − ***y***_2_)).

By framing both disjunctions and conjunctions in terms of phenotype multiplication, we can apply our established methods to approximate the covariances of these combinations with predictors and ultimately estimate linear models for these logical combinations.

While the case of the conjunction is a trivial application of the above methods of multiplying phenotypes, we will briefly describe how to model the disjunction. To do so, we consider the modified phenotypes $y_{1}^{\prime }=1_{n}-y_{1}$ and $y_{2}^{\prime }=1_{n}-y_{2}$ (these represent the statements “not ***y***_1_” and “not ***y***_2_”). This gives us ***y***_1_ ∨ ***y***_2_ = **1**_*n*_ − ***y***_1_′ ***y***_2_′. Then, ${\bar{y}}_{l}^{\prime }=1-{\bar{y}}_{l}$ , $s_{x_{j},y_{l}^{\prime }}=-s_{x_{j},y_{l}}$ , and $s_{y_{k}^{\prime },y_{l}^{\prime }}=s_{y_{k},y_{l}}$ . If we set $w_{2}^{\prime }={\bar{y}}_{1}^{\prime }{\bar{y}}_{2}^{\prime }$ , our method allow us to estimate $s_{x_{j},w_{2}^{\prime }}$ for each ***x***_*j*_ as well as ${\bar{w}}_{2}^{\prime }$ and $s_{w^{\prime }}^{2}$ . Leveraging these estimates, $s_{x_{j},w_{2}}=-s_{x_{j},w_{2}^{\prime }}$ , ${\bar{w}}_{2}=1-{\bar{w}}_{2}^{\prime }$ , and $s_{w_{2}}^{2}=s_{w_{2}^{\prime }}^{2}$ , where ***w***_2_ is equivalent to the disjunction ***y***_1_ ∨ ***y***_2_. Using these terms as inputs for the framework presented in Section 2.2 allow for coefficient and standard error estimation for the linear model $y_{1}\vee y_{2}=X\hat{\beta }+$ ϵ.

### 2.5 Simulation Studies

#### 2.5.1 Simulation 1: Type I Error Maintenance

To verify that our linear model with PCSS approach appropriately maintained the Type I error rate at a variety of *α* thresholds, we carried out a simulation under the null hypothesis that the predictor variant has no linear association with any of the phenotypes of interest. This null hypothesis represents a reasonable subset of the exact null hypothesis which is that the *product* of phenotypes has no linear relationship with the predictor. We carried out this simulation with varying sample size, MAF, phenotype means, phenotype correlations, and for continuous phenotypes, phenotype variances, for products of two binary phenotypes, two continuous phenotypes, and three continuous phenotypes. When simulating continuous phenotypes we assumed that $Y_{ik}∼N\left(\mu_{k},\sigma_{k}^{2}\right)$ with Cor(*Y*_*ik*_, *Y*_*il*_) *= ρ*_*kl*_ while when simulating binary phenotypes we let *Y*_*ik*_ ∼ Bernoulli*(μ*_*k*_ *)* (again with Cor(*Y*_*ik*_ *, Y*_*il*_ *) = ρ*_*kl*_). Simulation parameters (e.g., *n*, *μ*_1_, *ρ*_12_) were randomly sampled from various distributions (full details are available in Supplementary Table S1). We carried out 10^8^ simulations for each collection of continuous phenotypes and 10^7^ simulations for the case of binary phenotypes.

#### 2.5.2 Simulation 2: Comparisons to IPD Models

To evaluate our method’s ability to replicate the results of covariate adjusted linear models fit to IPD, we carried out three 2^*k*^ factorial simulations—one for the product of two binary phenotypes, one for the product of two positive continuous phenotypes, and one for the product of three positive continuous phenotypes. We carried out 1,000 simulations at each possible combination of parameters. In each simulation, we modeled the phenotype product as a function of a SNP and binary covariate. For the simulations with only two phenotypes, we also included a continuous covariate in our models.

In all simulations, we simulated *n* subjects’ SNP minor allele counts ***x***_1_ at HWE with varying MAF. We simulated a binary covariate ***x***_2_ ∼ Bernoulli(logit^−1^α_2_ ***x***_1_). When generating sets of two phenotypes we also generated a continuous covariate ***x***_3_ from a linear model with ***x***_1_ such that ${\bar{x}}_{3}=0$ , $s_{x_{3}}^{2}=1$ , and $r_{x_{1},x_{3}}=\alpha_{3}$ . This resulted in a SNP with two covariates (*p* = 3) in our two phenotype simulations, and a SNP with one covariate (*p* = 2) in our three phenotype simulation.

We generated individual phenotype measures through the model

$$u\left(y_{ik}\right)=\beta_{k0}+\overset{p}{\underset{j=1}{\sum }}x_{ij}\beta_{kj}+\epsilon_{ik}$$

where *u*(*y*_*ik*_) = *y*_*ik*_ for continuous phenotypes, *u*(*y*_*ik*_) = logit(Pr(*Y*_*ik*_ = 1)) for binary phenotypes, and ϵ_i_^′^ follows a multivariate normal distribution with μ=0 and **Σ**_(*i*,*j*)_ = *σ*_*i*_ *σ*_*j*_ *ρ*_*ij*_. Parameter values were selected such that, under optimal settings, empirical power was roughly 80–90*%* at a significance threshold of 10^−8^. Full details of simulation parameters are available in Supplementary Table S2.

In each simulation, we estimated coefficients, standard errors, *t* statistics, and two-sided *p*-values for the null hypothesis that there was no relationship between the product of phenotypes and the SNP (***x***_1_) after adjusting for covariates both using IPD and using PCSS.

Additionally, when simulating two binary phenotypes we fit covariate-adjusted logistic regression models for the logged odds that *y*_1*i*_ *y*_2*i*_ = 1 using IPD and returned the relevant two-sided *p*-value to compare the results of the linear model fit using PCSS to the correctly specified logistic model.

### 2.6 Real Data Application

Fatty acids are of broad importance for a wide range of cardiometabolic traits (Imamura et al., 2020) with ratios of fatty acids are often used as a proxy for conversion efficiency. Previous genome wide association studies have explored the genetic architecture of fatty acids and their ratios (Kalsbeek et al., 2018; Lemaitre et al., 2011; Tintle et al., 2015, 2020).

We modeled 12 fatty acid ratios using both IPD and PCSS using data from the Framingham Heart Study’s Generation-3 and Offspring cohorts downloaded from dbGaP (Mailman et al., 2007). The specific ratios can be found in the first column of Table 3. Supplementary Table S3 lists all fatty acids used in at least one of the ratios alongside their abbreviations.

Quality control measures included setting Mendelian inconsistencies as missing and excluding SNPs with HWE *p* < 0.00001, MAF <0.05, or missing values for over 10*%* of subjects. We excluded individuals missing over 10*%* of their genetic data after initial quality control and then took a subset of unrelated participants. After quality control we were left with 362,330 SNPs over 1,455 individuals (657 from the Offspring cohort and 888 from the Generation-3 cohort).

In addition to the standard PCSS described in Section 2.1, we assumed access to pre-computed means and variances of the reciprocal of each fatty acid as well as the correlation between any fatty acid reciprocal and any other fatty acid, covariate, or SNP to model these ratios using PCSS.

We analyzed each fatty acid ratio through the linear model: Ratio ∼ SNP + age + sex for each SNP in our sample using both IPD and PCSS and tested each SNP for statistical significance with the Bonferroni adjusted threshold *α* = 1.37 × 10^−7^.

### 2.7 Statistical Analysis

#### 2.7.1 Simulation 1

To analyze the results of our Type I Error simulations we calculated the empirical Type I Error rate when approximating linear models using PCSS at each specified significance threshold.

#### 2.7.2 Simulation 2

For each of the three 2^*k*^ factorial simulations, we assessed the PCSS model’s accuracy relative to its IPD counterpart.

We calculated the bias and mean squared error when estimating the SNP’s slope coefficient, standard error, and absolute value test statistic. We also modeled errors estimating the slope coefficient, standard error, and test statistic through multiple linear regression models with logical indicators for each of the *k* parameter settings as predictors, testing at the Bonferroni adjusted significance threshold of 0.05/*k* to determine which simulation parameters affected our method’s accuracy.

We compared test decisions regarding the significance of the SNP when modeling the phenotype product after adjusting for covariates at significance thresholds 10^−1^, 10^−2^, …, 10^−8^. When analyzing binary phenotypes we also compared test decisions between the linear model fit using PCSS and the logistic regression model fit on IPD to demonstrate the robustness of linear models to model binary outcomes.

#### 2.7.3 Real Data Application

We measured our overall bias and mean squared error in slope, standard error, and absolute value test statistic estimates for each outcome modeled. We recorded test decisions for both the IPD and PCSS models and whether the two results agreed or disagreed regarding the statistical significance of each SNP. When one approach found a SNP to be significant and the other did not, we noted if the non-significant result was “borderline” significant (*α* ≤ *p* < 10*α*).

### 2.8 Software

Software to perform these model approximations as well as those developed in Wolf et al. (2020) is available on CRAN in the R package pcsstools^5^.

## 3 Results

### 3.1 Simulation Studies

#### 3.1.1 Simulation 1

Empirical Type I error rates when using PCSS are displayed in Table 1. In all simulations, the approach’s empirical Type I error rate was below the tested significance threshold.

**TABLE 1:** Simulation studies of Type I error estimates when testing the linear association between a single SNP and a product of phenotypes using pre-computed summary statistics at significance thresholds: *α* = 0.05, 0.001, 10^−5^, and 10^−6^. Each entry represents the proportion of *p*-values smaller than *α* when modeling the linear relation between a SNP and a product of phenotypes.

	Nominal *α*			
Phenotypes	5.0E-02	1.0E-03	1.0E-05	1.0E-06
2 Continuous	3.88E-02	6.65E-04	5.56E-06	4.40E-07
2 Binary	2.39E-02	2.06E-04	8.00E-07	1.00E-07
3 Continuous	2.70E-02	3.81E-04	2.91E-06	3.40E-07

#### 3.1.2 Simulation 2

**FIGURE 2:**
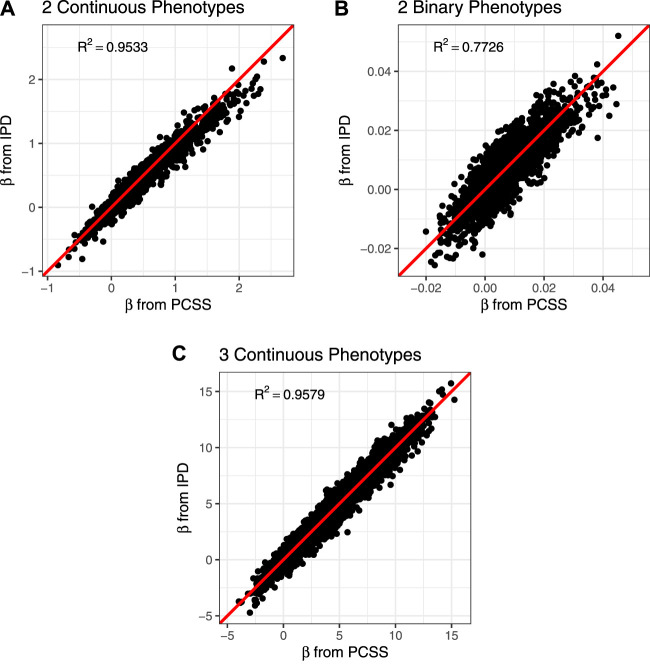
Comparison of slope coefficients from a simulation study approximating a covariate adjusted linear model for a product of phenotypes using pre-computed summary statistics (PCSS) and individual participant data (IPD). **(A)** Modeling the product of two continuous phenotypes while adjusting for a binary and a continuous covariate. **(B)** Modeling the product of two binary phenotypes while adjusting for a binary and a continuous covariate. **(C)** Modeling the product of three continuous phenotypes while adjusting for a binary covariate.

The PCSS method’s errors when approximating slope coefficients, their standard errors, and test statistics are available in Table 2. When aggregated over all simulation settings we observe slight positive bias both when estimating the slope and the absolute value of the *t* test statistic for each collection of phenotypes. The magnitude of the mean test statistic error is comparable across all three simulations. Figure 2 displays our PCSS method’s approximated slope coefficients compared to slope coefficients calculated using IPD for the SNP while modeling the phenotype product and adjusting for covariates. Similar graphical comparisons of standard error and test statistic estimates are available in Supplementary Figures S2, S3.

**TABLE 2:** Simulation study approximating a linear model for a product of phenotypes using summary statistics. Summaries of errors when approximating slopes, slope standard errors, and the absolute value of the *t*-statistics for a SNP while adjusting for covariates when using pre-computed summary statistics (PCSS) compared to values obtained when calculating these statistics using individual participant data (IPD).

Phenotypes	*β*			SE(*β*)		|*t*|	
	IPD Mean	Bias	MSE	Bias	MSE	Bias	MSE
2 Continuous	6.09E-03	3.72E-04	1.43E-05	2.39E-06	6.58E-11	2.33E-03	4.13E-01
2 Binary	4.13E-01	2.56E-02	6.74E-03	6.58E-05	5.50E-07	1.65E-01	2.16E-01
3 Continuous	4.82E+00	3.33E-02	3.79E-01	-3.63E-02	5.82E-04	5.71E-02	1.06E-01

When modeling estimation errors for two continuous phenotypes through a linear regression model with indicator variables for all of the simulation settings (*k* = 12, *n* = 2^*k*^ × 10^3^), our model for the slope error found all settings except the residual phenotype variances, $\sigma_{k}^{2}$ , to be significantly associated with the PCSS model’s slope estimate’s error at the adjusted significance threshold 0.05/*k*. All settings had significant associations with our error when estimating the standard error of the slope coefficient, or the test statistic. In the case of two binary phenotypes (*k* = 14, *n* = 2^*k*^ × 10^3^), we found all settings to have significant associations with the error in slope, standard error, and test statistic estimates. For three continuous phenotypes (*k* = 13, *n* = 2^*k*^ × 10^3^), we also found all settings to have significant associations with the error when predicting the slope coefficient, its standard error, and its test statistic.

**FIGURE 3:**
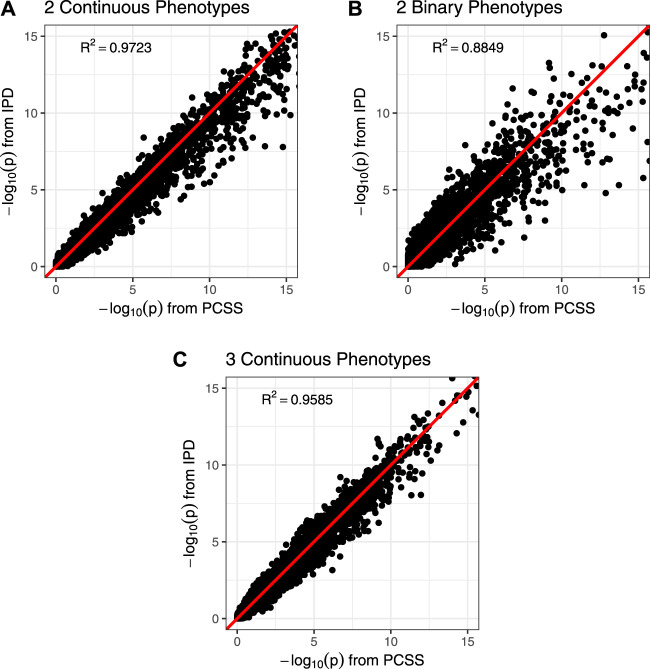
Comparison of *p*-values from a simulation study approximating a covariate adjusted linear model for a product of phenotypes using pre-computed summary statistics (PCSS) and individual participant data (IPD). Two-sided *p*-values were computed for the null hypothesis that the SNP had no linear effect on the phenotype product while adjusting for covariates. All plots are restricted to the range (0, 15); unrestricted plots are available in the supplementary materials. **(A)** Modeling the product of two continuous phenotypes while adjusting for a binary and a continuous covariate. **(B)** Modeling the product of two binary phenotypes while adjusting for a binary and a continuous covariate. **(C)** Modeling the product of three continuous phenotypes while adjusting for a binary covariate.

**FIGURE 4:**
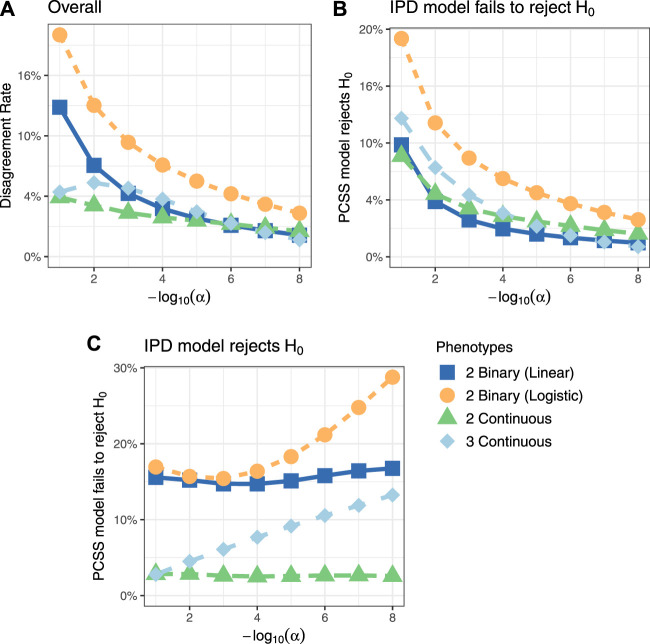
Simulation studies’ test decision disagreement rates evaluating the significance of a SNP in a linear model for a product of phenotypes while adjusting for covariates using individualized participant data (IPD) and pre-computed summary statistics (PCSS) at various significance thresholds (*α*). Comparisons were also made between a logistic regression model fit using IPD on the product of two binary phenotypes and the PCSS model approximating the linear relationship. **(A)** Percentage of times the PCSS and IPD models’ test decisions disagreed across all simulations. **(B)** Percentage of times the PCSS model rejected the null hypothesis given that the IPD model failed to reject the null hypothesis. **(C)** Percentage of times the PCSS model failed to reject the null hypothesis given that the IPD model rejected the null hypothesis.

Figure 3 shows comparisons of estimated and calculated *p*-values for a two-sided *t* test under the null hypothesis that the SNP had no linear association with the phenotype product after adjusting for covariates. (Figure 3 only includes *p*-values greater than 10^−15^ for the sake of visual clarity; Supplementary Figure S4 repeats this visualization without any restrictions.) Figure 4 shows various error rates rate between the IPD and PCSS models’ test decisions based on these *p*-values at differing significance thresholds. We see that all PCSS models overall disagreement rates to their IPD companions decrease as the significance threshold becomes more stringent. Likewise, when the IPD model rejected the null hypothesis, the PCSS model rarely failed to reject with error rates at most 13% which again decreased as the significance threshold decreased. When the IPD model failed to reject the null hypothesis, the PCSS approach’s conditional error rate varied by the model’s response. When modeling the product of two continuous or binary phenotypes, the error rate stayed relatively constant across all thresholds at around 3 and 15%, respectively. But, when modeling the product of three continuous phenotypes, the error rate increased as the significance threshold became more strict. Lastly, when compared to the test decisions of a covariate adjusted logistic regression model, our PCSS approximation of the related linear model tends to reach the same conclusions, with a moderate conservative tendency, especially at more strict significance thresholds.

### 3.2 Real Data Application

The bias and mean squared error of the PCSS model’s approximation to the IPD model’s slope, standard error, and absolute value test statistic for each fatty acid ratio are displayed in Table 3. Our mean slope error was −2.93 × 10^−3^ (Mean Squared Error 0.114) while the mean slope estimate when using IPD was −1.3 × 10^−3^, demonstrating a slight bias towards zero. However, the standard error estimates under the PCSS model were on average 4.80 × 10^−2^ lower than their respective estimates under the IPD model and absolute value tests statistics tended to be 1.79 × 10^−2^ higher than their IPD counterparts indicating an overall minor anti-conservative bias.

**TABLE 3:** Summary of errors when approximating the linear model: FA Ratio ∼ snp + age + sex using pre-computed summary statistics (PCSS) compared to values obtained when calculating these statistics using individual participant data (IPD). Each fatty acid ratio was modeled across 362,330 SNPs from 1,455 subjects in the Framing Heart Study’s Offspring and Generation-3 cohorts.

Ratio	*β*			SE(*β*)		|*t*|	
	IPD Mean	Bias	MSE	Bias	MSE	Bias	MSE
PA:POA	5.58E-02	1.49E-02	4.18E-02	−4.80E-02	2.08E-04	4.38E-03	1.45E-02
PA:SA	5.83E-05	−2.13E-05	3.09E-07	1.43E-04	1.87E-09	4.67E-03	8.23E-03
POA:OA	−2.08E-05	−2.10E-06	8.08E-09	−2.69E-05	4.69E-11	5.92E-03	1.70E-02
SA:OA	−8.15E-05	−1.19E-05	3.62E-07	−3.33E-05	3.36E-10	6.86E-03	8.21E-03
LA:GLA	−7.35E-02	2.03E-02	1.32E+00	−5.10E-01	1.95E-02	3.11E-02	3.17E-02
LA:DGLA	2.04E-03	1.99E-04	3.58E-04	−1.84E-03	3.12E-07	2.33E-02	4.08E-02
GLA:DGLA	4.58E-05	1.04E-05	2.64E-07	−7.62E-06	1.13E-10	3.36E-02	6.50E-02
DGLA:AA	−1.99E-05	−1.54E-06	2.84E-08	3.79E-05	1.09E-10	9.37E-03	1.33E-02
AA:DTA	1.76E-03	−4.20E-04	7.21E-05	−3.76E-03	9.42E-07	9.73E-03	2.38E-02
EPA:DPA_N3	2.29E-04	−1.38E-05	1.31E-06	−4.04E-04	1.02E-08	2.34E-02	4.72E-02
DTA:DPA_N6	−1.49E-03	1.66E-04	7.46E-04	−1.16E-02	8.24E-06	4.71E-02	1.43E-01
DPA_N3:DHA	−4.12E-04	6.03E-05	3.45E-06	−1.52E-04	2.53E-09	1.56E-02	3.75E-02
Overall	−1.30E-03	2.93E-03	1.14E-01	−4.80E-02	2.12E-02	1.79E-02	3.77E-02

Table 4 summarizes the number of SNPs found significant when modeling using both IPD and PCSS across all 12 × 362330 models. Of the ten SNPs for which IPD and PCSS models disagreed, nine occurred when one approach found a SNP to have a significant effect while the other found it to have a borderline significant effect (*α* ≤ *p* < 10*α*).

**TABLE 4:** Summary of test decisions for a real data application calculating the linear model Fatty Acid Ratio ∼ snp + age + sex using individual participant data (IPD) and pre-computed summary statistics (PCSS) across 362,330 SNPs from 1,455 subjects in the Framing Heart Study’s Offspring and Generation-3 cohorts. Significance threshold of *α* = 1.37 × 10^−7^.

Ratio	IPD significant	PCSS significant	Both significant
PA:POA	0	0	0
PA:SA	0	0	0
POA:OA	0	0	0
SA:OA	6	9	6
LA:GLA	5	2	2
LA:DGLA	9	10	9
GLA:DGLA	8	8	8
DGLA:AA	18	19	18
AA:DTA	0	0	0
EPA:DPA_N3	0	1	0
DTA:DPA_N6	5	4	4
DPA_N3:DHA	11	11	11
Overall	62	64	58

## 4 Discussion

We have developed a method that approximates the covariance of products of phenotypes with other variables using only bivariate and univariate pre-computed summary statistics (PCSS). We then demonstrated how this covariance estimation can be used to approximate linear models for products of phenotypes, how these can model logical “and” and “or” statements and how these models can include researchers choice of covariates. We demonstrated our approximation method’s accuracy relative to models fit on individual participant data through multiple simulations and applications to real genetic data.

The approximations showed good performance overall. In a wide variety of simulations, the Type I error was maintained, the bias in point estimates relative to models fit with IPD was minimal, and hypothesis tests almost always reached the same conclusion as would be obtained with IPD. Application of our method to real data from the Framingham Heart Study also showed good performance on similar metrics. In general, we have tried to formulate this PCSS method to only rely on commonly available or easily estimated PCSS. However, in our application we assumed that we had the PCSS for reciprocals of fatty acids. This may not always be the case in practice, but may suggest that these PCSS may be important to pre-compute to assist downstream analyses of ratios.

Despite these positive results, some limitations of our work are worth noting. First, we used linear regression for a binary response. Previous applications of PCSS have taken this approach (Canela-Xandri et al., 2018), and it is generally robust; however, this approach is less precise than when the underlying relationship is truly linear. While some foundations for a logistic modelling approach were recently proposed by Wu et al. (2021), further work is needed to develop a comprehensive model for logistic regression using PCSS. We also note that our method makes assumptions about the fit of the linear model to the data. While these assumptions are the same as in the corresponding analysis of IPD data (e.g., true underlying linear relationship between ***y*** and ***x***), these assumptions may be more acutely important in our PCSS method.

Second, while our simulation study was comprehensive and we demonstrated our method on real data we note that further testing on simulated and real data is encouraged to explore special cases not considered here. Situations that suggest further testing and methodological developments include modeling linear combinations of products and adjusting for clustered/family data. One scenario of particular importance that warrants further investigation is missing data resulting in PCSS being computed on different subsets of the full data. While our real data analysis showed good performance in a setting with missing genotype data, further investigations should be performed to address the robustness of this method (along with other methods based around PCSS) when dealing with missing phenotype information. Similarly, researchers may be limited in their ability to have additional *a priori* exclusion criteria applied to their analysis or obtain PCSS calculated using the same criteria for each phenotype. There are, however, two options. Either obtain summary statistics on the subgroup of interest or ignore the exclusion criteria by including the group in the “controls” in an analysis on a dichotomous outcome. More robust options are in development (e.g., multinomial regression, or methods that use summary statistics alone to allow researchers to apply exclusion criteria). Relatedly, while our method exhibited fair performance when modeling logical combinations of binary phenotypes with low case-control ratios (see Supplementary Table S4), it would benefit from further and more thorough work to assess its robustness.

Third, when estimating the variance of a product of a set of continuous phenotypes we estimated this term as a function of each covariate and took the median all these estimates as the approximated value. While this approach works well in practice, it may be possible to utilize the joint distribution of the covariates to estimate the covariance of these estimations and derive a more optimal estimation. Relatedly, our simulations showed that when multiplying binary phenotypes that exhibit high negative correlation and when multiplying phenotypes that take on negative values care should be taken. Finally and relatedly is the issue of potential compounding of errors when the method is applied to products of *m* phenotypes (where *m* is large). Although there are meaningful combined phenotypes that consist of five or more phenotypes (upon which this method should be used cautiously), we note that many combined phenotypes [e.g., BMI, ratios of biomarkers, cardiovascular disease (defined as either coronary heart disease or stroke)] are combinations of four or fewer phenotypes. Additional simulation studies and methodological improvements are needed and caution should be exhibited when applying our method in these cases.

Fourth and finally, this method does not support PCSS that describe score tests (where a null model with non-genetic covariates is first fit and then updated for each genetic variant instead of simultaneously estimating both the genetic and covariate effects) in its current form. While future research can likely expanded this method to work with this data, we note the considerable collection of PCSS repositories (e.g., PheWeb, GWAS Catalog, GeneAtlas) which do provide the PCSS needed to perform these proposed approximations.

The use of PCSS provides numerous advantages over IPD data including computational efficiency and reduced concerns about data privacy. However, substantially improved and flexible methods are needed in order to fully leverage PCSS in customized downstream analyses. Our method allows researchers further customization of analyzed phenotypes by opening the door to multiplicative combinations of phenotypes, including logical combinations of binary phenotypes. Approximations used are reasonable, with near perfect maintenance of the Type I error rate and power in most situations. Further work is needed to apply the method to additional datasets and to expand the method to larger classes of combined phenotypes.

## Data Availability

Publicly available datasets were analyzed in this study. This data can be found here: https://www.ncbi.nlm.nih.gov/gap/ (accessions phs000007.v29.p10 and phs000342.v20.p13).

## Footnotes

1
https://www.genome.gov/Funded-Programs-Projects/Electronic-Medical-Records-and-Genomics-Network-eMERGE

2
http://pheweb.sph.umich.edu/

3
http://pheweb.sph.umich.edu/

4
https://www.leelabsg.org/resources

5
https://cran.r-project.org/package=pcsstools
