# Supplementary material for: Using Summary Statistics to Model Multiplicative Combinations of Initially Analyzed Phenotypes With a Flexible Choice of Covariates
Source: Front Genet. 2021 Oct 12;12:745901. doi: 10.3389/fgene.2021.745901 (PMC8546319; doi:10.3389/fgene.2021.745901)
Supplement: Supplementary file 1 [file DataSheet1.pdf]

Supplementary Material for “Using summary statistics to model multiplicative combinations of initially analyzed phenotypes with a flexible choice of covariates”

1 FIGURES

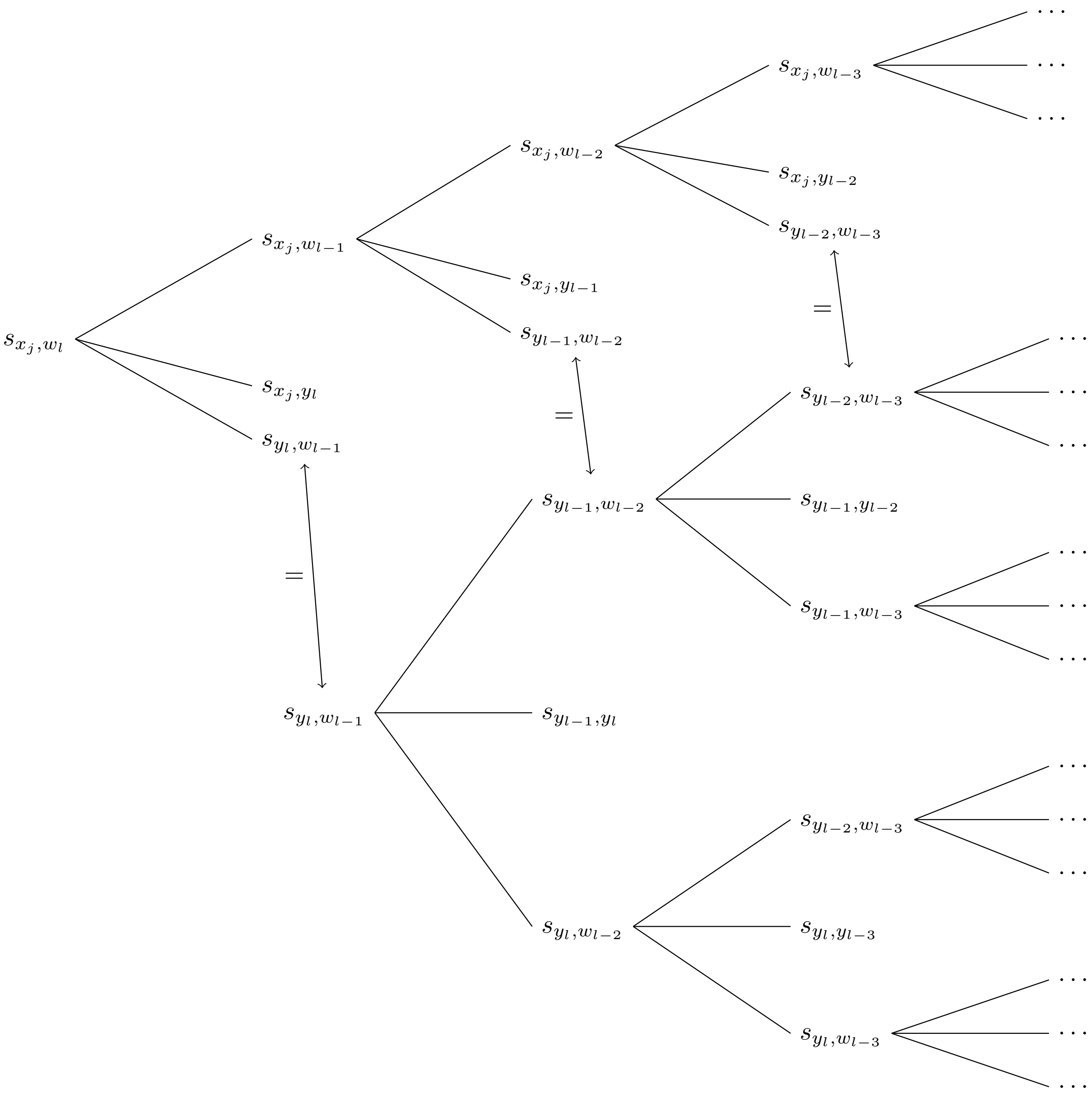

Figure S1. Diagram of the recursive algorithms used to approximate s_{x_j,w_l}. Three covariances are input (along with related means and variances) to approximate their parent node (to the left) using the method established in Section 2.3.1.

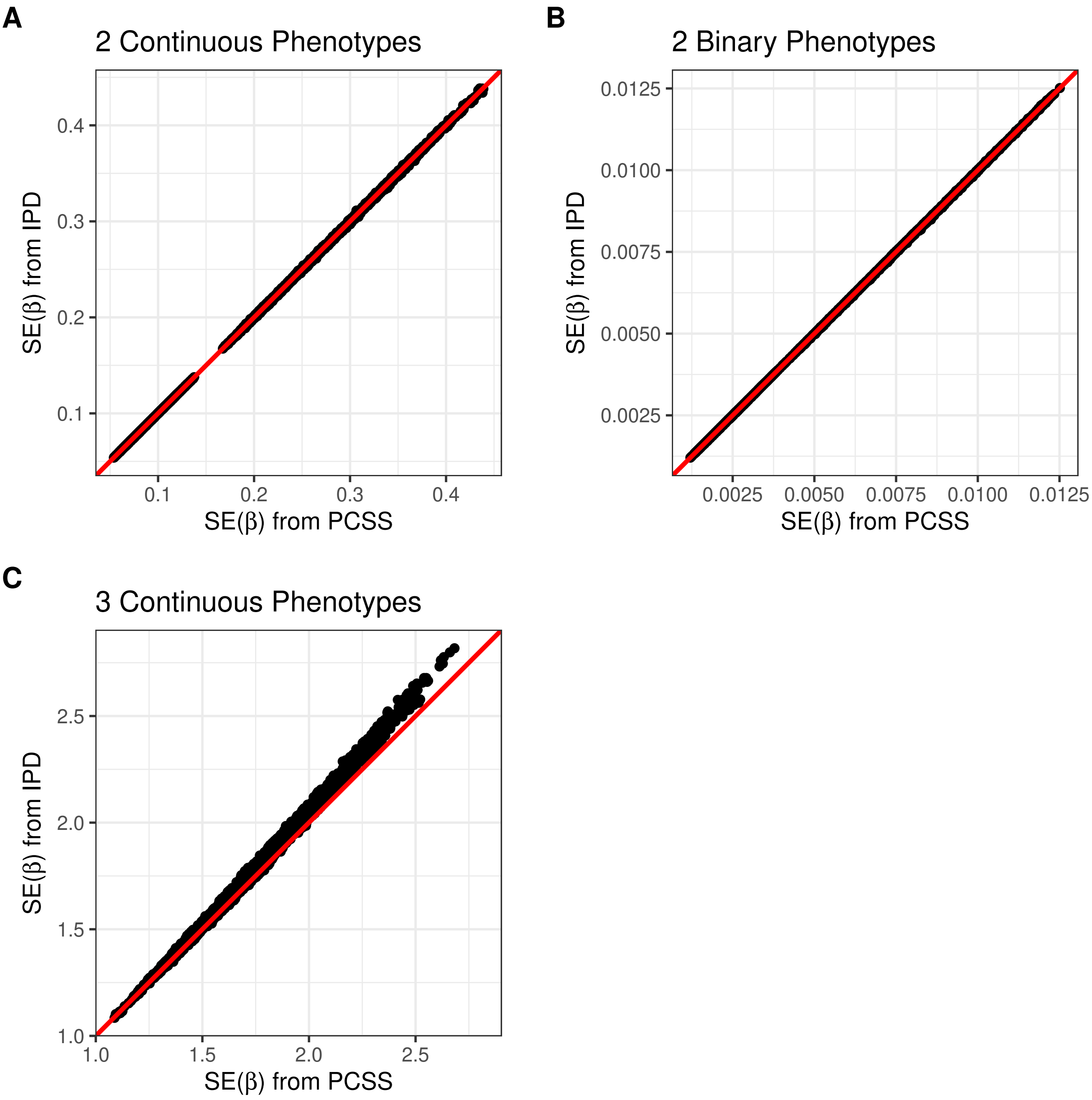

Figure S2. Comparison of slope coefficients' standard errors from a simulation study approximating a covariate adjusted linear model for a product of phenotypes using pre-computed summary statistics (PCSS) and individual participant data (IPD). **(A)** Modeling the product of two continuous phenotypes while adjusting for a binary and a continuous covariate. **(B)** Modeling the product of two binary phenotypes while adjusting for a binary and a continuous covariate. **(C)** Modeling the product of three continuous phenotypes while adjusting for a binary covariate.

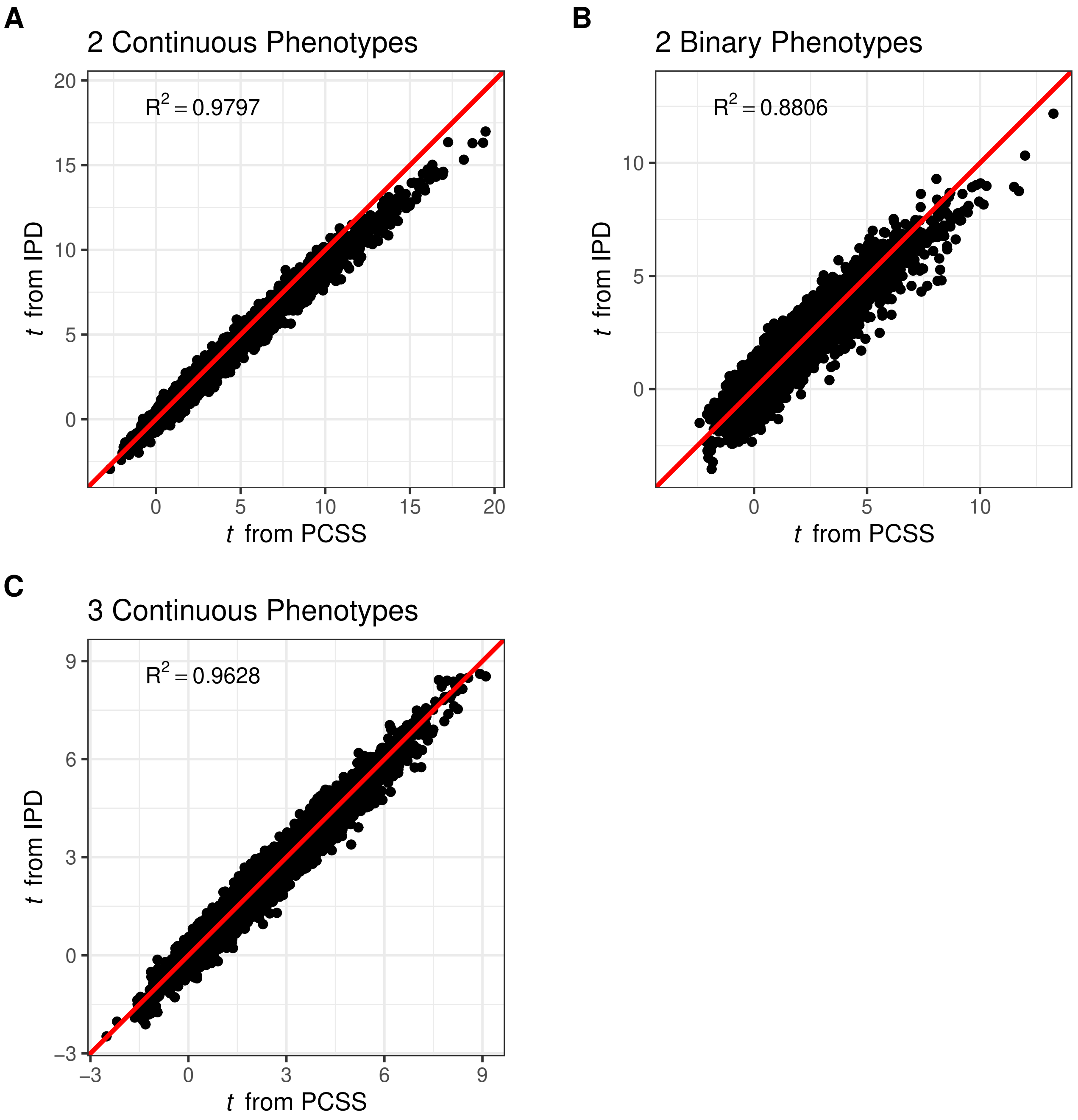

Figure S3. Comparison of t statistics from a simulation study approximating a covariate adjusted linear model for a product of phenotypes using pre-computed summary statistics (PCSS) and individual participant data (IPD). **(A)** Modeling the product of two continuous phenotypes while adjusting for a binary and a continuous covariate. **(B)** Modeling the product of two binary phenotypes while adjusting for a binary and a continuous covariate. **(C)** Modeling the product of three continuous phenotypes while adjusting for a binary covariate.

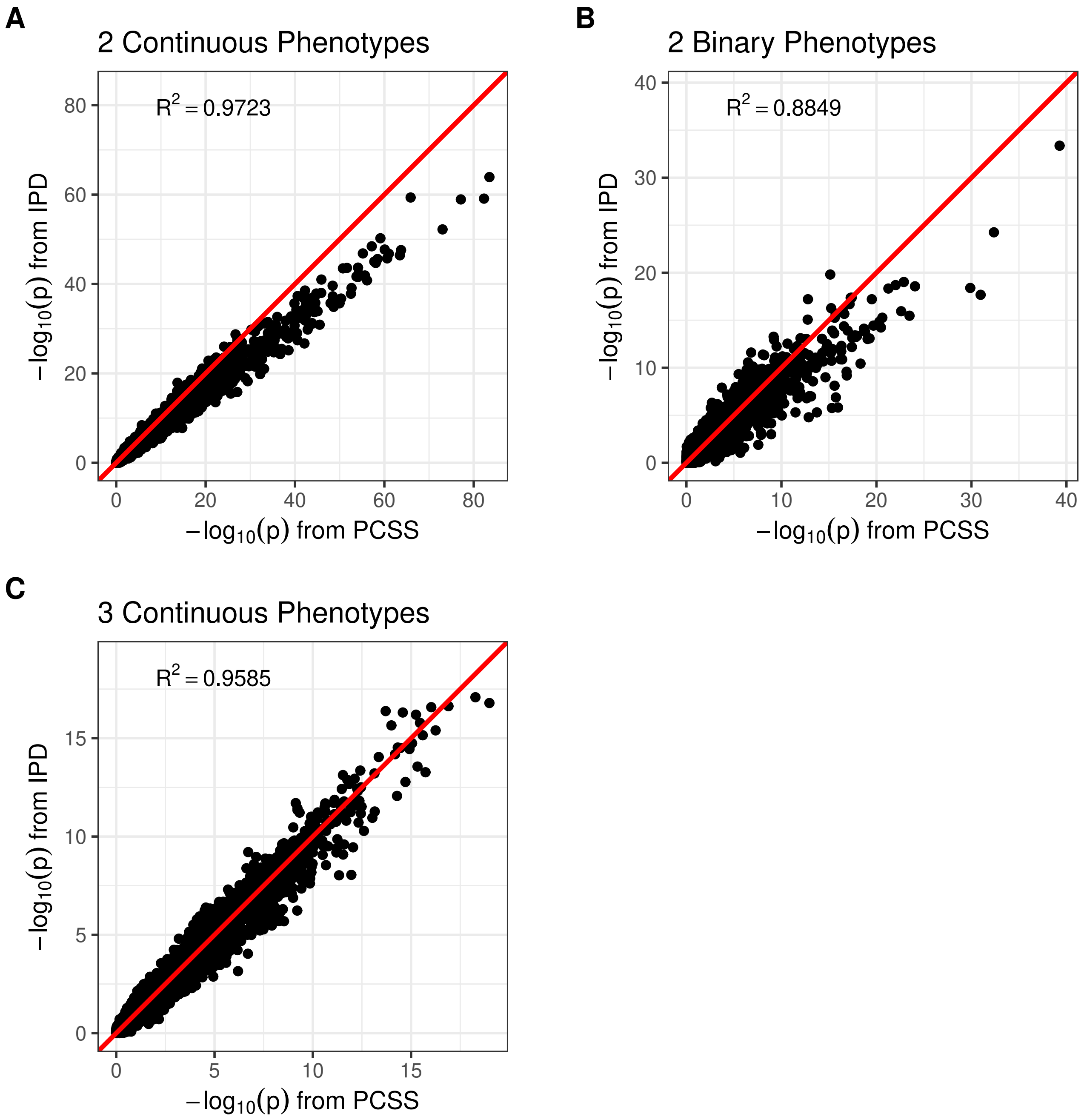

Figure S4. Comparison of p-values from a simulation study approximating a covariate adjusted linear model for a product of phenotypes using pre-computed summary statistics (PCSS) and individual participant data (IPD). Two-sided p-values were computed for the null hypothesis that the SNP had no linear effect on the phenotype product while adjusting for covariates. **(A)** Modeling the product of two continuous phenotypes while adjusting for a binary and a continuous covariate. **(B)** Modeling the product of two binary phenotypes while adjusting for a binary and a continuous covariate. **(C)** Modeling the product of three continuous phenotypes while adjusting for a binary covariate.

2 TABLES

Table S1. Distributions used to generate simulation parameters for the Type I error simulations. Continuous phenotypes were generated through a multivariate normal distribution while binary phenotypes were generated through a pair of correlated Bernoulli distributions. Correlations of two binary variables were simulated uniformly from the range of possible correlations for a given set of marginal probabilities μ_1 and μ_2 within the closed interval $[-0.25, 0.95]$.

	n	MAF	μ_k	σ_k	ρ_{kl}
Continuous	$10^4, 10^5$	$\mathrm{Unif}(0.05, 0.4)$	$N(0, 25)$	$\mathrm{Gamma}(1, 1/2)$	$\mathrm{Unif}(-0.5, 0.5)$
Binary			$\mathrm{Unif}(0.2, 0.8)$	NA	$\mathrm{Unif}(f(\mu_1, \mu_2))$

Table S2. Simulation parameters for 2^k factorial simulations. We carried out 1,000 simulations at each possible combination of settings for each set of phenotypes. Phenotype measures, or in the case of binary phenotypes logged odds of success, were simulated from a multivariate normal distribution conditional on variables $\boldsymbol{x}_1$, $\boldsymbol{x}_2$, and, when we generated only 2 phenotypes, $\boldsymbol{x}_3$. Parameters were selected such that the empirical power of models using individual participant data was around 90% under optimal settings. Columns with a value for Setting 1 but an "—" for Setting 2 indicate that the parameter was fixed at the value of Setting 1 in all simulations.

		n	MAF	α_2	α_3	β_{k0}	β_{k1}	β_{k2}	β_{k3}	σ_k^2	ρ_{kl}
2 Continuous	Setting 1	10^4	0.10	0	0	5	0.01	0.1	0.1	2	0
$(k = 12)$	Setting 2	10^5	0.25	$\ln 2$	0.4	—	0.05	1	1	3	0.4
2 Binary	Setting 1	10^4	0.01	0	0	0	$\ln 1.001$	0.01	0.01	1	0
$(k = 14)$	Setting 2	10^5	0.25	$\ln 2$	0.4	$\ln 1/4$	$\ln 1.075$	$\ln 2$	$\ln 2$	5	0.4
3 Continuous	Setting 1	10^4	0.15	0	NA	5	0.01	0.1	NA	1.5	0
$(k = 13)$	Setting 2	—	—	$\ln 2$	NA	—	0.10	1	NA	3	0.4

Table S3. Fatty acids in at least one analyzed ratio with abbreviations.

Fatty Acid	Abbreviation	SHARE Variable Name
Palmitic Acid	PA	RBC_C16_0
Stearic Acid	SA	RBC_C18_0
Palmitoleic Acid	POA	RBC_C16_1
Oleic Acid	OA	RBC_C18_1
Eicosapentaenoic Acid	EPA	RBC_C20_5N3
Docosapentaenoic Acid n-3	DPA_N3	RBC_C22_5N3
Docosahexaenoic Acid	DHA	RBC_C22_6N3
Linoleic Acid	LA	RBC_C18_2N6
Gamma-linolenic Acid	GLA	RBC_C18_3N6
Dihomo-gamma-linoleic Acid	DGLA	RBC_C20_3N6
Arachidonic Acid	AA	RBC_C20_4N6
Docosapentaenoic Acid-n6	DPA_N6	RBC_C22_5N6
Docosatetranoic Acid	DTA	RBC_C22_4N6

Table S4. Simulation study assessing the affect of different case-control ratios on the performance of estimation of covariate adjusted linear models for the product of two binary phenotypes using pre-computed summary statistics. Covariate adjusted linear regression models were fit for either $\boldsymbol{y}_1 \wedge \boldsymbol{y}_2$ or $\boldsymbol{y}_1 \vee \boldsymbol{y}_2$ (respectively representing "and" and "or" statements). Data were generated via the model $\text{logit}(\Pr(Y_{ik} = 1)) = \text{logit}(p_k) + \beta_{k1}(x_{i1} - \bar{x}_1)/s_{x_1} + \beta_{k2}x_{i2}$ for SNP x_{i1} with MAF 0.2 and standard normal covariate $\boldsymbol{x}_2$ which was independent of $\boldsymbol{x}_1$. We fixed $n = 5000$ and $\beta_{k2} = \log(1.01)$ and let $p_k \approx \Pr(Y_{ik} = 1)$ take on values 0.01, 0.05, 0.10, and let β_{k1} be either $\log(1.01)$ or $\log(1.10)$. We carried out 1000 simulations for each combination of simulation parameters. Reported values are aggregated across all combinations of β_{11} and β_{21}.

		β			SE(β)		$\|t\|$	
p_1	p_2	IPD Mean	Bias	MSE	Bias	MSE	Bias	MSE
$\boldsymbol{y}_1 \wedge \boldsymbol{y}_2$								
0.01	0.01	2.04E-05	-1.60E-06	6.62E-08	8.09E-08	1.47E-14	-7.70E-01	8.69E-01
0.01	0.05	9.81E-05	-1.64E-06	3.27E-07	1.12E-07	3.03E-14	-6.15E-01	7.56E-01
0.01	0.10	1.94E-04	3.82E-06	6.10E-07	1.44E-07	5.30E-14	-4.83E-01	6.76E-01
0.05	0.01	9.82E-05	7.29E-06	3.06E-07	1.06E-07	2.75E-14	-5.94E-01	7.28E-01
0.05	0.05	4.72E-04	2.80E-05	1.58E-06	2.34E-07	1.45E-13	-4.51E-01	6.97E-01
0.05	0.10	9.26E-04	-4.32E-05	2.87E-06	3.17E-07	2.97E-13	-3.67E-01	6.60E-01
0.10	0.01	1.93E-04	-1.90E-05	6.30E-07	1.52E-07	5.97E-14	-5.15E-01	7.23E-01
0.10	0.05	9.12E-04	-1.97E-05	2.95E-06	3.23E-07	3.01E-13	-3.79E-01	6.84E-01
0.10	0.10	1.78E-03	-1.86E-05	5.55E-06	4.22E-07	6.65E-13	-2.73E-01	6.52E-01
$\boldsymbol{y}_1 \vee \boldsymbol{y}_2$								
0.01	0.01	1.97E-03	2.36E-06	6.85E-08	4.95E-09	8.44E-15	-2.63E-03	5.45E-03
0.01	0.05	5.47E-03	4.93E-06	3.31E-07	1.06E-08	4.92E-14	-1.92E-03	9.25E-03
0.01	0.10	9.36E-03	-5.19E-06	6.43E-07	1.16E-08	1.17E-13	-2.43E-03	1.04E-02
0.05	0.01	5.55E-03	9.83E-06	3.33E-07	5.47E-09	4.83E-14	2.38E-05	9.34E-03
0.05	0.05	8.99E-03	1.32E-05	1.61E-06	3.41E-08	2.78E-13	2.82E-04	2.83E-02
0.05	0.10	1.24E-02	3.24E-05	2.89E-06	5.98E-08	5.87E-13	-3.57E-03	3.65E-02
0.10	0.01	9.52E-03	-5.62E-06	6.31E-07	1.33E-08	1.20E-13	-1.45E-03	1.02E-02
0.10	0.05	1.24E-02	2.59E-05	3.03E-06	4.95E-08	6.21E-13	-3.36E-03	3.81E-02
0.10	0.10	1.57E-02	4.83E-05	5.80E-06	8.52E-08	1.31E-12	-3.43E-03	5.85E-02
